# Transcriptome profiling unveils the role of cholesterol in IL-17A signaling in psoriasis

**DOI:** 10.1038/srep19295

**Published:** 2016-01-19

**Authors:** Pallavi Varshney, Aarti Narasimhan, Shankila Mittal, Garima Malik, Kabir Sardana, Neeru Saini

**Affiliations:** 1 Functional Genomics Unit, CSIR - Institute of Genomics and Integrative Biology, Mall Road, Delhi-110007, India.; 2 Academy of Scientific & Innovative Research, CSIR-Institute of Genomics and Integrative Biology, Delhi 110007, India; 3 Department of Dermatology, Maulana Azad Medical College, New Delhi.

## Abstract

Psoriasis is a chronic inflammatory skin disease characterized by altered proliferation and differentiation of keratinocytes as well as infiltration of immune cells. Increased expression of Th17 cells and cytokines secreted by them provides evidence for its central role in the pathogenesis of psoriasis. IL-17A, signature cytokine of Th17 cells was found to be highly differentially expressed in psoriatic lesional skin. However, cellular and molecular mechanism by which IL-17A exerts its function on keratinocyte is incompletely understood. To understand IL-17A mediated signal transduction pathways, gene expression profiling was done and differentially expressed genes were analysed by IPA software. Here, we demonstrate that during IL-17A signaling total cholesterol levels were elevated, which in turn resulted in the suppression of genes of cholesterol and fatty acid biosynthesis. We found that accumulation of cholesterol was essential for IL-17A signaling as reduced total cholesterol levels by methyl β cyclodextrin (MBCD), significantly decreased IL-17A induced secretion of CCL20, IL-8 and S100A7 from the keratinocytes. To our knowledge this study for the first time unveils that high level of intracellular cholesterol plays a crucial role in IL-17A signaling in keratinocytes and may explain the strong association between psoriasis and dyslipidemia.

Psoriasis is a chronic inflammatory skin disease with 2–3% prevalence worldwide. It is characterized by the erythematosquamous plaques, formed due to hyper proliferation and abnormal differentiation of keratinocytes as well as infiltration of immune cells^1^. This disease has an incompletely defined etiology. Currently there is no cure, but efficacious treatment to control its symptoms exists. Multiple genome wide linkage studies revealed the association of polymorphisms in keratinocyte differentiation genes and the genes encoding the components of immune system with psoriasis^2^. Increasing epidemiological studies suggest that psoriasis patients are at significantly higher risk of developing cardiovascular and metabolic diseases. Systemic inflammation is expected to be the cause of the association of psoriasis with metabolic diseases and this association increases with the increasing disease severity^3^,^4^.

Involvement of the immune system in psoriasis is now widely accepted. Psoriasis is defined as a Th1/Th17 based inflammatory disease^5^. It involves a crosstalk between immune cells including dendritic and T-cells with keratinocytes^6^. Naive T cells differentiate into Th17 cells in the presence of IL-6, IL-23 and TGF-β cytokines derived from antigen presenting cells^7^. On activation Th17 cells produce several mediators such as IL-24, IL-21, IL-22, TNF-α, IL-17 which induce keratinocyte proliferation and other hallmark features of psoriasis. IL-23/ Th-17 axis is thought to be a central player in psoriatic inflammation as genetic susceptibility to psoriasis is linked to IL-23p19 and IL-23p40 gene variants and to the IL-23 receptor^8^,^9^. Literature reveals high levels of Th17 cell-specific cytokines IL-17A and IL-22 in psoriasis^10^,^11^,^12^. Moreover, monoclonal antibodies directed against IL23p40 have shown beneficial effects in psoriasis patients and thus it is predicted that inhibition of IL-23 will abort Th17 cell differentiation and result in abrogation of psoriasis.

In the current study, we sought to compare different cytokine and growth factor levels in the skin biopsies of psoriatic patients and reported IL-17A as highly differentially expressed cytokine in the psoriatic lesional skin. Moreover, we identified the critical role of IL-17A in regulating sterol regulatory element binding proteins (SREBPs), a family of transcription factors that regulate lipid homeostasis. We herein describe a previously unidentified mode of action of IL-17A potentially opening new avenues for the treatment of psoriasis.

## Results

### Cytokine expression profiling in psoriatic lesional and non-lesional skin

**Figure 1:**
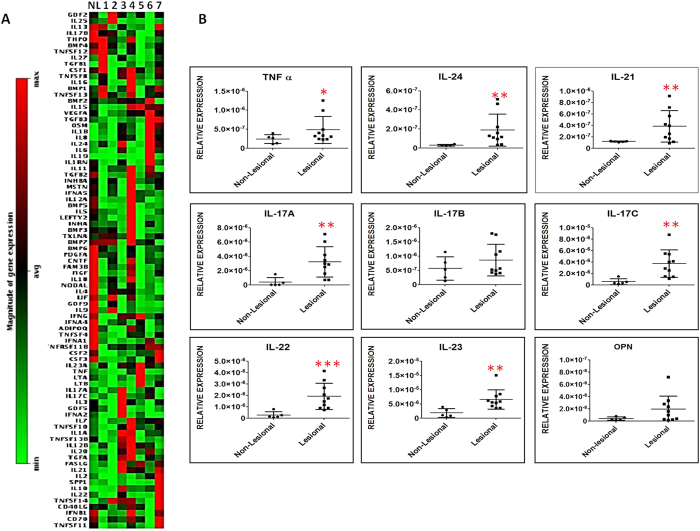
Cytokine profiling in psoriatic lesional and non-lesional epidermis. (**A**) Cytokine PCR array for lesional (1–7) and non-lesional (NL- average of expression values of 4 tissues) skin biopsies. Heat map depicts the relative expression values in psoriatic lesional (n = 7) and non- lesional skin (n = 4) for the 84 cytokines. **(B)** Expression of Th17 related cytokines including TNF-α, IL-24, IL-21, IL-17A, IL-17B, IL-17C, IL-22, IL-23 and IL-16 were measured by Q-RT PCR and represented as a dot plot of relative expression (Delta Ct) values for both non-lesional (n = 5) and lesional skin (n=10). Significant ***p-value (≤0.001), **p-value (≤0.01), *p-value (≤0.05) was calculated using Student’s two-tailed t test.

To gain insight into the cytokines involved in psoriasis pathogenesis, expression of 84 different cytokines were assessed by RT^2^ profiler PCR array in the psoriatic lesional and non-lesional skin. Supplementary Table S1 shows the information of total patients enrolled in this study (Biopsies taken from top 7 subjects marked in red were used for cytokine expression profiling and other biopsies were used for validation purposes. Heat map (Fig. 1A) depicts the relative expression values for the 84 cytokines in psoriatic lesional (n = 7) and non-lesional skin (n = 4). Consistent with the previous reports (2), we found an increase in the expression of several Th17 related cytokines in psoriatic subjects.

Comparison of gene expression for the cytokines secreted by Th17 cells (IL-24, IL-21, IL-22, TNF-α, IL-17A), cytokines involved in Th17 differentiation (IL-23, IL-21) and cytokines regulating Th17 cytokine response (OPN) and different members of IL-17 family (IL-17A, IL-17B, IL-17C) were further done by quantitative real time PCR (Q-RT PCR). As expected, we found significant up regulation of TNF-α (2 fold), IL-24 (9 fold), IL-21 (3.5 fold), IL-17A (11.5 fold), IL-17B (1.5 fold), IL-17C (6.6 fold), IL-22 (9.5 fold), IL-23 (3.6 fold) and OPN (4 fold) in the psoriatic lesional skin as compared to non lesional skin (Fig. 1B). Among all these, we found highest differential expression of IL-17A in the psoriatic lesional skin. Given that IL-17A is associated with a number of auto-immune diseases in humans, dissecting its molecular mechanism in keratinocytes will help in understanding its impact in psoriasis.

### IL-17A induced alterations in gene expression and their functional classification in primary human keratinocytes

**Figure 2:**
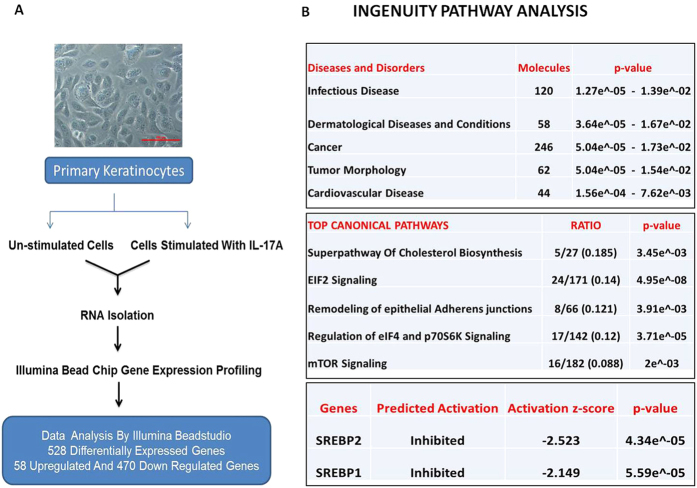
Gene expression profiling in IL-17A stimulated human primary keratinocytes and Ingenuity Pathway Analysis (IPA) summary. (**A**) Schema for Illumina Gene expression profiling on un-stimulated and IL-17A stimulated primary keratinocytes at 6h and data was average normalized and list of 528 differentially expressed genes were obtained with detection p-value (≤0.01) and differential p-value of (≤0.05). **(B)** List of Top five diseases and canonical pathways along with their respective ratio and p-values obtained from IPA analysis. Two key transcription factors SREBP1 and SREBP2 found to have a negative activation z-score as calculated by IPA.

In order to understand the cellular and molecular mechanism of IL-17A function in keratinocytes we captured early events in this signaling. Using an Illumina platform, genome wide gene expression profiling was carried out in primary normal human epidermal keratinocytes (NHEK) stimulated for 6h with IL-17A (Fig. 2A.). Analysis of the microarray data using Bead studio software revealed 528 differentially expressed genes (DEG, detection p-value ≤ 0.01; differential p-value ≤ 0.05) shown in Supplementary Table S3 and out of which 58 genes were found to be up-regulated and 470 to be down-regulated post IL-17A stimulation of keratinocytes.

Molecular and cellular functional analysis of DEG was done using Ingenuity pathway analysis (IPA Tool; Ingenuity Systems, Redwood City, CA, USA; http://www.ingenuity.com). In IPA, DEG were mapped to the genetic networks available in the Ingenuity database and associated with “Disease and Disorders” as shown in Fig. 2B. IPA analysis also determines the topmost “Canonical Pathways” represented in DEG along with the ratio. Ratio denotes the number of molecules from the gene list falling in a particular pathway divided by the total number of molecules present in that pathway in their database. Superpathway of cholesterol biosynthesis was found to have the highest ratio of 0.185 and with no previously known co-relation with IL-17A cytokine in the literature.

Literature reveals that cholesterol and fatty acid synthesis is regulated by SREBP family of transcriptional factors. SREBP2 regulates genes involved in cholesterol synthesis via HMGCR (3-hydroxy 3-methylglutaryl CoA reductase) and SREBP1 regulates fatty acid synthesis genes via FASN (Fatty acid synthase) and ACACA (Acetyl CoA carboxylase)^13^. Interestingly, IPA analysis further showed the activation z-score of SREBP2 as (−2.5) and SREBP1 as (−2.15). Activation z-score predicts the activation states of upstream transcriptional regulators (z-score < −2 explains the inhibited state of a molecule). All these results strongly suggest the role of IL-17A in regulating cholesterol and fatty acid metabolism in human keratinocytes.

### Effect of IL-17A signaling on cholesterol levels in keratinocytes and psoriatic skin

**Figure 3:**
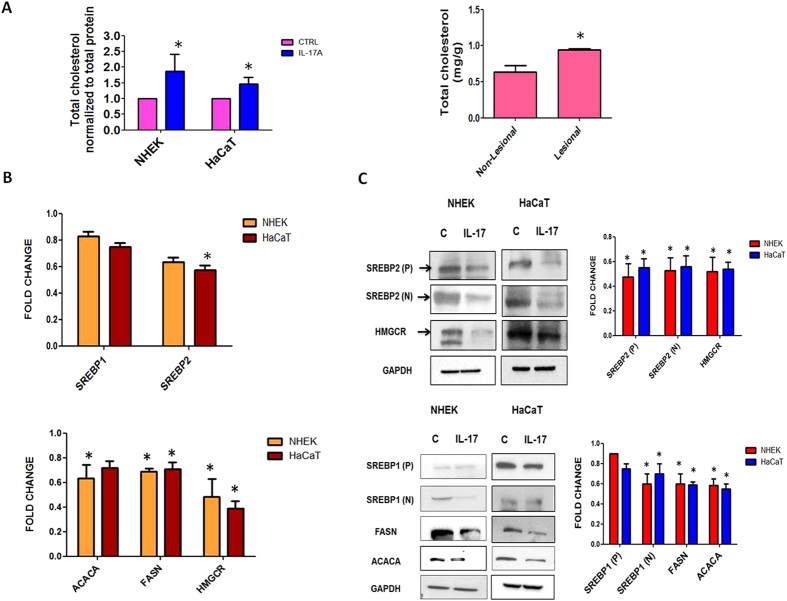
Validation of Micro-array data in human primary keratinocytes (NHEK) and HaCaT. (**A**) Estimation of total cholesterol content in IL-17A stimulated keratinocytes NHEK and HaCaT cells (normalized to total protein content) as well as in psoriatic lesional and non-lesional skin (normalized to tissue weight). **(B)** Real Time PCR analysis to determine the expression of SREBP transcriptional factors as well as their downstream genes HMGCR, FASN and ACACA in keratinocytes post IL-17A stimulation. The graph represents fold change in gene expression normalized to 18s rRNA expression. **(C)** Western blot analysis for the expression of genes involved in cholesterol biosynthesis (SREBP2, HMGCR) and fatty acid synthesis (SREBP1, FASN, ACACA) upon IL-17A treatment in keratinocytes. GAPDH was used as a loading control for densitometric analysis. Graph represents the fold change in protein expression compared to unstimulated cells. The data is expressed as the mean ± S.D. of 3 independent experiments. * indicates p-value (≤0.05) in comparison to unstimulated cells.

To further confirm our above findings, we measured total cholesterol levels in primary keratinocytes and HaCaT cells post IL-17A stimulation (6h) using cholesterol estimation kit. As shown in Fig. 3A, we found significant higher levels of cholesterol (2 fold) in NHEK and (1.5 fold) HaCaT cells respectively. Moreover, we found decreased cholesterol levels when IL-17A signaling was inhibited by IL-17A neutralising antibody in HaCaT cells as shown in Supplementary Fig. S1. We next found increased levels of cholesterol in psoriatic lesional skin compared to non-lesional skin, which could be due to the elevated levels of IL-17A in the lesions of psoriatic patients. Here, we report that IL-17A signaling leads to cholesterol accumulation in keratinocytes which might be playing a crucial role in psoriasis pathophysiology.

### Validation of differentially expressed genes at transcriptional and translational levels

SREBPs regulate genes of cholesterol biosynthesis pathway and cholesterol levels in turn regulate the SREBP activity by a feedback mechanism. SREBPs in their inactive state remains bound to the ER. During low sterol levels they undergo specific proteolytic cleavage and the cleaved molecule translocates to the nucleus to initiate downstream gene expression^14^. Herein, we analysed the transcript levels of SREBPs in IL-17A stimulated keratinocytes NHEK and HaCaT by quantitative real time PCR and found a significant decrease in SREBP2 (1.6 fold) whereas insignificant changes in SREBP1 levels. Simultaneously, we found a significant decrease in HMGCR (2 fold), FASN (1.5 fold) and ACACA (1.6 fold) transcript levels respectively (Fig. 3B). We next found ≥2 fold decrease in the precursor (P) and cleaved form (N) of SREBP2 and HMGCR in both the cell types post IL-17A stimulation. Although there was no change in the expression of the precursor form of SREBP1 (P) but there was a significant decrease of 1.5–1.6 fold in the cleaved form of SREBP1 (N) in NHEK and HaCaT cells post IL-17A treatment. Significant decrease (1.6-1.5 fold) in the protein levels of FASN and ACACA were found in both the cell types (Fig. 3C). Taken together, all these findings demonstrate that IL-17A signaling leads to increased cellular cholesterol levels which in turn by a feedback mechanism down-regulates the expression of cholesterol and fatty acid biosynthesis genes at both transcriptional and translational levels.

### Altered expression of cholesterol and fatty acid biosynthesis genes in psoriatic skin

**Figure 4:**
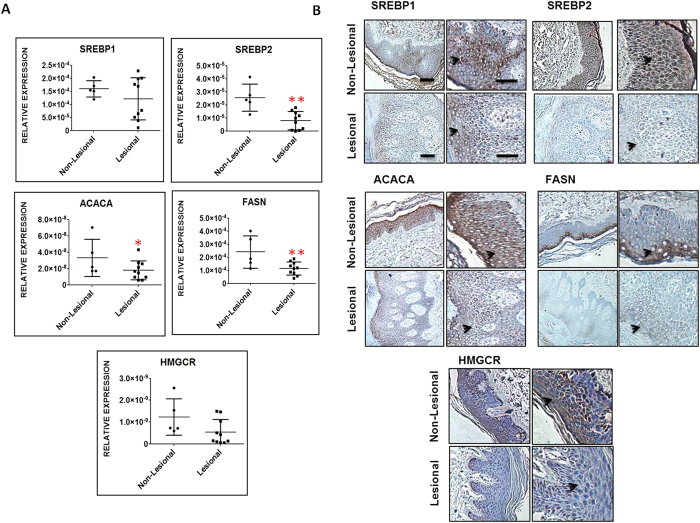
Analysis of cholesterol and fatty acid synthesis related gene expression in psoriatic lesional and non-lesional epidermis. (**A**) Q-RT PCR analysis of SREBP1, SREBP2, FASN, ACACA and HMGCR expression in nonlesional (n = 5) and lesional psoriatic skin (n = 10) and data is represented as a dot plot of relative expression (∆ Ct) values with significant **p-value (≤0.01) and *p-value (≤0.05). **(B)** Immunohistochemical staining of SREBP1, SREBP2, ACACA, FASN and HMGCR in psoriatic lesional and non-lesional skin tissues. Enlarged images of each staining are shown in the adjacent figure. Scale bar = 100 um.

As we observed significant up-regulation of IL-17A in psoriatic lesions, we next analysed the expression of SREBPs and its downstream molecules FASN, ACACA and HMGCR in the psoriatic skin. Q-RT PCR analysis showed significantly lower levels (>3fold) of SREBP2 in lesional skin compared to non-lesional while changes in the levels of SREBP1 were insignificant. Further, significant decrease (2.2 fold) in the levels of ACACA and FASN whereas insignificant changes in HMGCR levels were found in lesional skin compared to non-lesional skin (Fig. 4A).To further strengthen our findings, immunohistochemistry was performed on the psoriatic skin tissues (Fig. 4B) and the levels of SREBP1, SREBP2, ACACA, FASN and HGGCR were observed to be downregulated in the lesional epidermis compared to non-lesional skin. FASN expression was restricted to stratum lucidium and granulosum of the non-lesional epidermis and was found to have decreased expression in the lesional skin. Here, we confirm the decreased expression of SREBPs, HMGCR, FASN and ACACA in the psoriatic lesional skin consistent with the *in-vitro* IL-17A stimulated keratinocyte data.

### Effect of cholesterol alteration on IL-17A signaling

**Figure 5:**
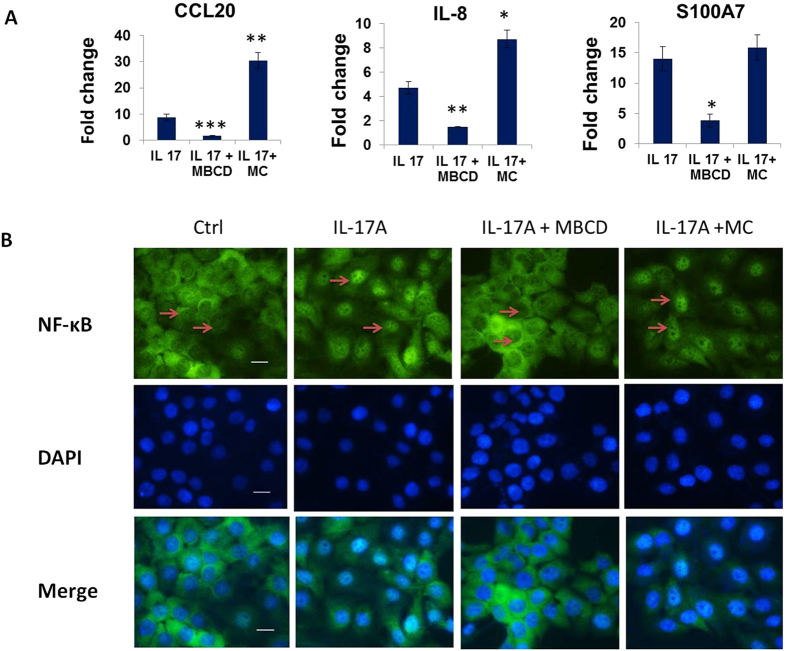
Cellular cholesterol depletion inhibits IL-17A signaling. (**A**) Analysis of CCL20, IL-8 and S100A7 expression in cholesterol depleted (MBCD) and replenished (MC) cells post IL-17A stimulation by Q-RT PCR. Graph represents the fold change in gene expression compared to unstimulated cells. The data is expressed as the mean ± S.D. of 3 independent experiments with ***p-value (≤0.001), **p-value (≤0.01), *p-value (≤0.05). **(B)** Localization of NF-κB p65 visualized 1h post IL-17A stimulation in cholesterol depleted and replenished HaCaT cells under fluorescence microscope after immunofluorescence staining with NF-κB p65 antibody (green). In addition, the cells were stained with DAPI to visualize nuclei (blue). Scale bar = 50 um.

Literature reveals that IL-17A stimulate keratinocytes to secrete chemokines (CCL20), pro-inflammatory cytokine (IL-8) and anti-microbial peptide (S100A7) via NF-κB activation which plays an important role in the pathogenesis of psoriasis^15^. To elucidate the role of increased cholesterol in IL-17A signaling, expression of CCL20, IL-8 and S100A7 was analysed by quantitative real time PCR in cholesterol depleleted and replenished cells. Cholesterol depletion was achieved by methyl-β cyclodextrin (MBCD)^16^ and repletion by MBCD-Cholesterol treatment for 1 h which was verified by filipin staining (Supplementary Fig. S2). Filipin binds to cholesterol with high affinity and is used as a histochemical stain for cholesterol^17^. MBCD treatment resulted in a reduced fluorescence intensity of filipin stain as compared with untreated cells. Conversely, MBCD-cholesterol resulted in enhanced fluorescence intensity. We found 5 fold decrease in CCL20, 3 fold decrease in IL-8 and S100A7 expression in cholesterol depleted cells and significant upregulation of these genes in cholesterol enriched cells post IL-17A stimulation (Fig. 5A). Inhibition of IL-17A signaling by neutralising antibody also showed decreased expression of these genes (Supplementary Fig. S3) similar to that observed in IL-17A stimulated cholesterol depleted cells. Our data clearly suggest that IL-17A mediate its effect through elevated cholesterol levels as decreasing cellular cholesterol inhibited cytokine signaling. Next, we also analysed the changes in IL-17A induced NF-κB activation by its nuclear translocation assay and found decreased NF-κB nuclear translocation in IL-17A stimulated cholesterol depleted cells while increased translocation in cholesterol replenished cells (Fig. 5B). All these data suggest that, IL-17A induced elevated cellular cholesterol plays a crucial role in the secretion of chemokines, pro-inflammatory cytokines and anti-microbial peptides via NF-κB activation from the keratinocytes.

## Discussion

Earlier Th1 cells were known to play a role in the initiation and maintenance of psoriatic phenotypes. Over the past few years, focus has shifted towards the involvement and role of Th17 cells in psoriasis. Among various Th17 related cytokines, we found elevated levels of IL-17A in the psoriatic lesional skin consistent with the previous reports.

In psoriatic skin, IL-17A is produced by CD4+ T cells, epidermal CD8+ T cells, neutrophils, mast cells, and macrophages 18. In keratinocytes, IL-17A induces secretion of anti-microbial peptides and chemokines like CCL20, CXCL1, CXCL2, CXCL3, CXCL5 via NF-κB and CCAAT/enhancer binding proteins (C/EBP) transcription factors^19^ which further recruits dendritic cells, Th17 cells and neutrophils at the site of a lesion^15^. IL-17A acts synergistically with other cytokines such as IL-22^20^ and TNF-α^21^ to augment the inflammatory response.

**Figure 6:**
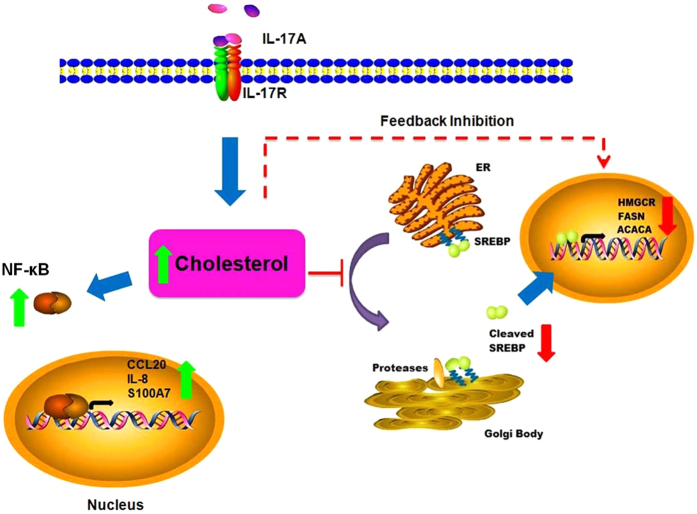
Central role of cholesterol homeostasis in IL-17A function. IL-17A stimulated keratinocytes showed elevated levels of cellular cholesterol. This increased cellular cholesterol by a feedback mechanism may be involved in inhibiting the cholesterol and fatty acid synthesis machinery via SREBP family of transcriptional factors. Elevated cholesterol also regulates the expression of CCL20, IL-8 and S100A7 in keratinocytes via activation of NF-κB and thus may play a crucial role in IL-17A induced psoriatic inflammation.

Herein, we found that IL-17A boosts intracellular cholesterol levels which in turn by a feedback mechanism inhibits the expression of SREBP2 and SREBP1 as well as their downstream target genes including HMGCR, FASN and ACACA (Fig. 6). Similar to our findings, several reports suggest an association of IL-17A with lipid metabolic disorders like atherosclerosis. IL-17A is considered to be proatherogenic and is found to be involved in the formation of foamy macrophages^22^. Recently Salvatore *et al.* found that IL-17A turned monocyte derived dendritic cells into foamy dendritic cells by increased cholesterol uptake^23^. Here, *in-vitro* studies on keratinocytes were further validated on psoriatic skin and we found elevated levels of cholesterol in psoriatic lesional skin and decreased expression of cholesterol and fatty acid related genes. Our studies were consistent with previous reports of increased total lipids, phospholipids, triacylglycerols, and cholesterol in both blood and epidermis of psoriatic patients^24^,^25^.

Herein, we report that cholesterol accumulation is essential for IL-17A signaling as cholesterol depletion resulted in decreased expression of CCL20, IL-8 and S100A7 via inhibiting NF-κB activation as shown in Fig. 6. Elevated cholesterol levels by IL-17A may be a reason for subclinical alterations of the epidermal skin in psoriasis. Similar to our findings, previous reports do suggest that the increased cellular cholesterol plays a role in enhanced secretion of pro-inflammatory cytokines^26^. Our study thus strengthens the link between inflammatory cytokines and cholesterol which would further help in understanding the role of elevated cholesterol in psoriasis pathophysiology.

Large number of epidemiological studies suggests strong association of psoriasis with dyslipidemia. Wu *et al.* recently showed that hypercholesterolemia is associated with an increased risk of psoriasis, particularly in those people who have hypercholesterolemia for more than 7 years^27^. Significant differences have also been observed in lipoprotein composition (HDL/LDL levels), HDL particle size, and cholesterol efflux mechanisms in patients with psoriasis. Successful treatment of psoriasis results in recovery of HDL particle size and cholesterol efflux capacity^28^,^29^. Athough a few theories have been proposed, there has been unsatisfactory explanation for dyslipidemia. Common T cell mediated inflammatory pathways are likely to be involved in the pathophysiology of psoriasis and cardiovascular inflammation, both of which are associated with a chronic proinflammatory, proangiogenic, and prothrombotic state^30^,^31^. Increased levels of intracellular cholesterol may affect the blood cholesterol levels and thus explain the low level of HDL and increased levels of LDL observed in psoriatic patients.

To the best of our knowledge this study for the first time unveils that high levels of intracellular cholesterol plays a crucial role in IL-17A induced signaling in keratinocytes and may explain the epidemiological evidence of strong association between psoriasis and dyslipidemia. Beneficial effect of statins used for the psoriatic treatment ^32^could be due to the inhibition of IL-17A signaling.

## Materials and Methods

### Cell cultures

HaCaT cell line was procured from National Centre for Cell Sciences (NCCS), Pune, India and maintained in DMEM F-12 media containing 10% (v/v) fetal calf serum, 100 Units/ml penicillin, 100mg/ml streptomycin, 0.25mg/ml amphotericin at 37 °C in a humidified atmosphere at 5% CO_2_. Primary normal human epidermal keratinocytes (NHEK) were isolated from the foreskin samples by 15–18 h treatment with Dispase II at 4 °C followed by trypsinization at 37 °C for 5 mins by 0.1% trypsin. Keratinocytes were cultured in keratinocyte serum free media supplemented with epidermal growth factor and bovine pituitary extract and once 80% confluent, cells were stimulated with recombinant human (rh) IL-17A (R&D System, Minneapolis, MN) at 100 ng/ml for 6 hours before harvesting for further experiments.

### Study design and patient entry criteria

Patients were screened from Outdoor Patient Department of the Department of Dermatology, Maulana Azad Medical College, and New Delhi (OPD) and were enrolled in the study based on certain inclusion and exclusion criteria. Inclusion criteria were patients of psoriasis aged ≥12 years having undergone no previous treatment for psoriasis. Patients with any present history of any other ailment were excluded from the study. Twenty four patients with varied PASI (psoriasis area severity index) score (2–50) were taken for the study and none of the patients were on statins prior to biopsy. Written informed consent was taken from all the subjects before recruitment in the study. Study was approved by the Human ethics committee of CSIR–Institute of Genomics and Integrative Biology. All experiments were performed in accordance with the approved guidelines. Punch biopsy of 4mm was taken from the site of psoriatic lesion as well as the non-lesional skin of the same patient.

### Human cytokine PCR array and quantitative real time PCR

For human cytokine PCR array total RNA was isolated from lesional and non-lesional psoriatic skin tissue using miRvana miRNA isolation kit (Ambion). Cytokine expression profiling of 500 ng total RNA purified from skin biopsies was done using RT^2^ SYBR Green Master Mix (Qiagen) and RT^2^ Profiler PCR Array Human Common Cytokines; PAHS-021ZC-12.

Quantitative real time PCR was carried out using cDNA synthesized by RevertAid H Minus firststrand cDNA synthesis kit as per the manufacturer’s instructions (Fermentas,Glen Burnie, MD, USA) and SYBR Green PCR master mix (Applied Biosystems, Foster City, CA, USA) in an ABI Prism 7500 Sequence Detection System (Applied Biosystems) and experiment was performed in triplicate and repeated thrice. Results were normalized with 18S rRNA and fold change was calculated using the Pfaffl’s method^33^. Primers used for the detection of expression levels of genes TNF-α, IL-24, IL-21, IL-17A, IL-17B, IL-17C, IL-22, IL-23, OPN, SREBP1, SREBP2, HMGCR, FASN, ACACA and 18s rRNA are listed in Supplementary Table S2.

### Illumina Microarray, data analysis and Pathway analysis:

Microarray was performed using human HT-12 v4 expression bead chip as described earlier^34^. The data was average normalized and FDR calculation was done using illumine (Beadstudio 2.0) custom test. The genes which crossed the threshold of detection p-value ≤0.05 and differential score p-value ≤ 0.05 were considered to be differentially expressed. Ingenuity Pathway Analysis software (IPA Tool; Ingenuity Systems, Redwood City, CA USA; http://www.ingenuity.com) was used to explore possible biological interactions of differentially expressed genes.

### Cholesterol content measurement

Cholesterol was quantified using cholesterol quantitation kit (Biovision, CA, USA) as described^35^. Briefly cells were subjected to extraction with organic solvents (7:11:0.1, chloroform/isopropanol/Triton X-100) and total cholesterol levels were measured by spectrophotometer at 570 wavelength and normalized to protein concentrations.

### Western Blotting

Western blotting was performed as described^36^ using unstimulated and stimulated cells with IL 17A after 6 h of stimulation. All the primary antibodies used for this work were obtained from commercial sources. SREBP2, SREBP1, ACACA and HMGCR from Abcam (Cambridge, MA, USA) and FASN and GAPDH Santa Cruz (Santa Cruz Biotechnology, Santa Cruz, CA, USA). The secondary antibodies were HRP-linked and blots were developed using enhanced chemiluminiscence (Thermo Scientific, Waltham, MA, USA).

### Immuno-histochemistry

Immunohistochemistry was performed using ultra-vision quanto detection kit as per the manufacturer’s instructions. Briefly, skin biopsies were fixed with 10% formalin, dehydrated, and embedded in paraffin. Sections were placed on polylysine-coated slides, deparaffinized, hydrated, antigen retrieved with 10 mM citrate buffer pH 6.0, and blocked with protein block for 10 mins followed by incubation with monoclonal antibody to SREBP1 (1:100), SREBP2 (1:100), FASN (1:100) and ACACA (1:250) overnight at 4 °C. Slides were incubated with HRP-polymer quanto for 30 minutes at room temperature followed by development using DAB quanto substrate. The sections were counterstained with hematoxylin and slides analysed by microscopy. Negative controls consisted of sections with no primary antibody treatment.

### Immunofluorescence

HaCaT cells were seeded and grown on cover slips in 6-well tissue culture plates. Next day, cells were treated with 70mM MBCD and MBCD-cholesterol^37^ for 1 h and then replaced with fresh media having 100 ng/ml IL-17A. After 6 h, cells were fixed (4% formaldehyde for 15 mins), permeabilized (0.1% Triton X-100 for 15 mins) followed by blocking with (1% BSA and 0.1% Triton X-100 in 1X PBS) for 1 h and then primary antibody NF-κB p65 (santa cruz) incubation overnigh. It was followed by FITC-conjugated secondary antibody (Invitrogen) for 1 h and then stained with DAPI for 20 min at room temperature. The fluorescence images were captured using Nikon microscope (Ti Eclipse).

### Statistical Analysis

Results are given as means of three independent experiments ± s.d. An independent Student’s two-tailed t test was performed using replicate values and significant ***p-value (≤0.001), **p-value (≤0.01), *p-value (≤0.05) was calculated.

## Additional Information

**How to cite this article**: Varshney, P. *et al.* Transcriptome profiling unveils the role of cholesterol in IL-17A signaling in psoriasis. *Sci. Rep.* **6**, 19295; doi: 10.1038/srep19295 (2016).

## Supplementary Material

Supplementary Information
