# Supplementary material for: Transcriptome profiling unveils the role of cholesterol in IL-17A signaling in psoriasis
Source: Sci Rep. 2016 Jan 19;6:19295. doi: 10.1038/srep19295 (PMC4726068; doi:10.1038/srep19295)
Supplement: Supplementary Information [file srep19295-s1.pdf]

Supplementary Data

Transcriptome profiling unveils the role of cholesterol in IL-17A signaling in psoriasis

Pallavi Varshney[1,2], Aarti Narasimhan[1], Shankila Mittal[3], Garima Malik[1], Kabir Sardana[3], Neeru Saini [1,2*]

[1]Functional Genomics Unit, CSIR - Institute of Genomics and Integrative Biology, Mall Road, Delhi-110007, India.

[2]Academy of Scientific & Innovative Research, CSIR-Institute of Genomics and Integrative Biology, Delhi 110007, India

[3] Department of Dermatology, Maulana Azad Medical College, New Delhi.

*Correspondence: Dr. Neeru Saini,
CSIR- Institute of Genomics and Integrative Biology,
Mall Road, Delhi- 110007, India.
Telephone: 91-11-27666156;
FAX: 91-11-27667471;
e-mail – nsaini@igib.in

Supplementary Table S1

S.No	AGE	SEX	PASI SCORE	SITE OF BIOPSY		ONSET AGE (YEARS)
				LESIONAL	NON-LESIONAL	
1	32	M	18.1	BACK	LOWER LEG	15
2	35	M	25.3	EXTENSOR FOREARM	-	35
3	28	F	18.6	KNEES	-	12
4	50	M	2.2	BACK	BACK	38
5	12	M	3.4	EXTENSOR FOREARM	FOREARM	9
6	45	M	46.4	ELBOWS	-	45
7	50	M	16.1	ELBOWS	FOREARM	50
8	24	M	14.8	EXTENSOR FOREARM	FOREARM	20
9	60	F	12.3	EXTENSOR FOREARM	FOREARM	50
10	55	M	14.9	BACK	-	30
11	24	M	35.3	EXTENSOR FOREARM	-	24
12	25	M	12.6	BACK	BACK	21
13	55	M	36.4	ANKLE	-	53
14	19	M	17	ELBOWS	-	15
15	32	F	12.1	EXTENSOR FOREARM	FOREARM	18
16	19	M	6.3	KNEES	LOWER LEG	19
17	65	M	6.2	BACK	BACK	40
18	62	M	46.4	BACK	-	55
19	24	M	9.1	ANKLE	LOWER LEG	20
20	40	F	4.4	KNEES	LOWER LEG	38
21	65	M	24	EXTENSOR FOREARM	-	62
22	42	M	31.5	EXTENSOR FOREARM	-	35
23	35	M	15.2	EXTENSOR FOREARM	-	33
24	58	M	25.6	EXTENSOR FOREARM	-	52

Table S1- Clinico-pathological details of patients - 24 patients suffering from psoriasis aged $\geq$ 12 years with varied PASI (psoriasis area severity index) score (2-50) and with no ongoing treatment for psoriasis were screened and enrolled in the study. Punch biopsy of 4mm was taken from lesional and non-lesional site of the same psoriatic patient.

Supplementary Table S2:

Gene	Primer sequence
IL-22 FP	CCAGCCTTATATGCAGGAGG
IL-22 RP	TTTCAGCTTTGCTCTGGTCA
OPN FP	CCAAGTAAGTCCAACGAAAG
OPN RP	GGTGATGCTCTCGTCTGTA
IL-17A FP	ACTACAACCGATCCACCTCAC
IL-17A RP	ACTTTGCCTCCCAGATCACAG
IL-23 FP	AGCCGCCCGGGTCTT
IL-23 RP	TCCTTGAGCTGCTGCCTTTAG
TNF-α FP	TTCTGTCTACTGAACTTCGGGGTGATCGGTCC
TNF-α RP	GTATGAGATAGCAAATCGGCTGACGGTGTGGG
IL-24 FP	GACTTTAGCCAGCAGACCCTT
IL-24 RP	GGTTGCAGTTGTGACACGAT
IL-21 FP	ATCCAGTCCTGGCAACATGG
IL-21 RP	GGGCCTTCTGAAAGCAGGAA
IL-17C FP	TCACCCTGGAGATACCGTGT
IL-17C RP	CGTGCATCGATACAGCCTCT
IL-17B FP	GGAATGGACTGGCCTCACAA
IL-17B RP	GGGTCGTGGTTGATGCTGTA
HMGCR FP	TCGGTGGCCTCTAGTGAGAT
HMGCR RP	TGTCCCCACTATGACTTCCC
FASN FP	CAGAGTCGGAGAACTTGCAG
FASN RP	GGAGGCATCAAACCTAGACAG
ACACA FP	AATCTTGAGGGCTAGGTCTTTCTGGA
ACACA RP	CCAGAGGTTGGGCCAAGGGA
SREBP1 FP	GCAAGGCCATCGACTACATT
SREBP1 RP	GGTCAGTGTGTCCTCCACCT
SREBP2 FP	AGGAGAACATGGTGCTGA
SREBP2 RP	TAAAGGAGAGGCACAGGA
CCL 20 FP	ATGTGCTGTACCAAGAGTTT
CCL 20 RP	CAAGTCTGTTTTGGATTTGC
IL-8 FP	GGCACAAACTTTCAGAGACAG
IL-8 RP	ACACAGAGCTGCAGAAATCAGG
S100A7 FP	AAC TTC CTT AGTGCC TGTG
S100A7 RP	TGG TAGTCTGTG GCT ATGTC
18S FP	GTAACCCGTTGAACCCCATT
18S RP	CCATCCAATCGGTAGTAGCG

Table S2 - 5’-3’ Primer sequences used for Real-time PCR (FP- Forward Primer, RP- Reverse Primer)

Supplementary Table S3

Gene symbol	Definition	Fold Change
AASDHPPT	Aminoadipate-Semialdehyde Dehydrogenase-Phosphopantetheinyl Transferase	0.501312348
ABCC4	ATP-Binding Cassette, Sub-Family C (CFTR/MRP), Member 4	0.578768361
ABHD15	Abhydrolase Domain Containing 15	0.284287929
ACCN2	Amiloride-Sensitive Cation Channel 2, Neuronal	0.416026796
ACSL1	Acyl-Coa Synthetase Long-Chain Family Member 1	0.227768644
ACTR3	ARP3 Actin-Related Protein 3 Homolog (Yeast)	0.14725208
ADA	Adenosine Deaminase	1.524166873
ADAM8	ADAM Metallopeptidase Domain 8	2.319511096
ADD3	Adducin 3 (Gamma)	0.492170775
ADNP	Activity-Dependent Neuroprotector Homeobox	0.53450135
AGFG1	Arfgap With FG Repeats 1	0.419182509
AIDA	Hypothetical LOC653631; Hypothetical LOC646050; Hypothetical LOC646890; Axin Interactor, Dorsalization Associated	0.546870287
AIM1	Absent In Melanoma 1	0.526625057
AK2P2	Adenylate Kinase 2 Pseudogene 2	0.473461259
AK3L1	Adenylate Kinase 3-Like 2; Adenylate Kinase 3-Like 1	0.470909365
AKAP11	A Kinase (PRKA) Anchor Protein 11	0.567662525
ALCAM	Hypothetical Protein LOC100133690; Activated Leukocyte Cell Adhesion Molecule	0.609719365
ALDH1B1	Aldehyde Dehydrogenase 1 Family, Member B1	0.290508661
ALG5	Asparagine-Linked Glycosylation 5, Dolichyl-Phosphate Beta-Glucosyltransferase Homolog (S. Cerevisiae)	0.319880617
AMMECR1L	AMME Chromosomal Region Gene 1-Like	0.410932689
AMY1C	Amylase, Alpha 1A (Salivary); Amylase, Alpha 1B (Salivary); Amylase, Alpha 1C (Salivary)	0.378289253
ANKRD27	Ankyrin Repeat Domain 27 (VPS9 Domain)	0.295693573
ANKRD30B	Ankyrin Repeat Domain 30B	0.483590368
ANO4	Anoctamin 4	0.285640318
ANXA2P1	Annexin A2 Pseudogene 3; Annexin A2; Annexin A2 Pseudogene 1	0.19904008
ANXA2P3	Annexin A2 Pseudogene 3; Annexin A2; Annexin A2 Pseudogene 1	0.405673559
ANXA3	Annexin A3	0.332464001
ANXA4	Annexin A4	0.038609387
ANXA8	Annexin A8	0.468533929
ANXA8L1	Annexin A8-Like 1	0.562763504
APPL1	Adaptor Protein, Phosphotyrosine Interaction, PH Domain And Leucine Zipper Containing 1	0.458459009
ARL16	ADP-Ribosylation Factor-Like 16	0.479939104
ARPC5	Actin Related Protein 2/3 Complex, Subunit 5, 16kda	0.443324134
ARPP19	Camp-Regulated Phosphoprotein 19 Pseudogene; Camp-Regulated Phosphoprotein, 19kda	0.480848205

**ATF1**	**Activating Transcription Factor 1**	**0.169649329**
**ATP11B**	**Atpase, Class VI, Type 11B**	**0.486679119**
**ATP1B3**	**Atpase, Na+/K+ Transporting, Beta 3 Polypeptide**	**0.478986788**
**ATP5C1**	**ATP Synthase, H+ Transporting, Mitochondrial F1 Complex, Gamma Polypeptide 1**	**0.456404973**
**AVEN**	**Apoptosis, Caspase Activation Inhibitor**	**0.533894576**
**B4GALT1**	**UDP-Gal:Betaglcnac Beta 1,4- Galactosyltransferase, Polypeptide 1**	**0.547129949**
**BCHE**	**Butyrylcholinesterase**	**0.147831831**
**BLVRB**	**Biliverdin Reductase B (Flavin Reductase (NADPH))**	**0.243482503**
**BLZF1**	**Basic Leucine Zipper Nuclear Factor 1**	**0.34809154**
**BRI3P1**	**Brain Protein I3; Brain Protein I3 Pseudogene 1**	**0.252260935**
**BTG2**	**BTG Family, Member 2**	**2.230406434**
**C10orf137**	**Chromosome 10 Open Reading Frame 137**	**0.50608254**
**C10orf88**	**Chromosome 10 Open Reading Frame 88**	**0.427960859**
**C12orf23**	**Chromosome 12 Open Reading Frame 23**	**0.019447842**
**C12orf48**	**Chromosome 12 Open Reading Frame 48**	**0.487190338**
**C13orf27**	**Chromosome 13 Open Reading Frame 27**	**0.359072996**
**C14orf106**	**Chromosome 14 Open Reading Frame 106**	**1.703427139**
**C14orf166**	**Chromosome 14 Open Reading Frame 166**	**0.50375354**
**C17orf91**	**Chromosome 17 Open Reading Frame 91**	**0.442656307**
**C18orf32**	**Chromosome 18 Open Reading Frame 32**	**0.181796511**
**C1GALT1**	**Core 1 Synthase, Glycoprotein-N-Acetylgalactosamine 3-Beta-Galactosyltransferase, 1**	**0.142010719**
**C1orf212**	**Chromosome 1 Open Reading Frame 212**	**0.288916328**
**C1orf63**	**Chromosome 1 Open Reading Frame 63**	**0.542524304**
**C20orf108**	**Chromosome 20 Open Reading Frame 108**	**0.382037042**
**C5orf28**	**Chromosome 5 Open Reading Frame 28**	**0.262523213**
**C6orf115**	**Chromosome 6 Open Reading Frame 115**	**0.547150284**
**C6orf173**	**Chromosome 6 Open Reading Frame 173**	**0.380145038**
**C7orf49**	**Chromosome 7 Open Reading Frame 49**	**0.550912012**
**C7orf55**	**Chromosome 7 Open Reading Frame 55**	**1.714805123**
**C8orf59**	**Chromosome 8 Open Reading Frame 59**	**0.514760598**
**C9orf130**	**Chromosome 9 Open Reading Frame 130**	**0.413212989**
**C9orf82**	**Chromosome 9 Open Reading Frame 82**	**0.257052497**
**C9orf85**	**Chromosome 9 Open Reading Frame 85**	**0.386798462**
**CAPZA1**	**Capping Protein (Actin Filament) Muscle Z-Line, Alpha 1**	**0.207611269**
**CARS2**	**Cysteinyl-Trna Synthetase 2, Mitochondrial (Putative)**	**0.494102804**
**CASP6**	**Caspase 6, Apoptosis-Related Cysteine Peptidase**	**0.536010762**
**CAST**	**Calpastatin**	**0.370053256**
**CAT**	**Catalase**	**0.603142672**
**CCDC104**	**Coiled-Coil Domain Containing 104**	**0.432149941**
**CCDC107**	**Coiled-Coil Domain Containing 107**	**2.396476437**
**CCDC125**	**Coiled-Coil Domain Containing 125**	**0.297959416**
**CCDC132**	**Coiled-Coil Domain Containing 132**	**0.144907472**
**CCDC43**	**Coiled-Coil Domain Containing 43**	**0.545952232**

**CCNG2**	**Cyclin G2**	**0.413385902**
**CCT6A**	**Chaperonin Containing TCP1, Subunit 6A (Zeta 1)**	**0.509924619**
**CCT6P1**	**Chaperonin Containing TCP1, Subunit 6 (Zeta) Pseudogene 1**	**0.352863495**
**CD164**	**CD164 Molecule, Sialomucin**	**0.000421042**
**CD68**	**CD68 Molecule**	**0.400207537**
**CDC5L**	**CDC5 Cell Division Cycle 5-Like (S. Pombe)**	**0.262034445**
**CGN**	**Cingulin**	**2.698555865**
**CHD1**	**Chromodomain Helicase DNA Binding Protein 1**	**0.442452479**
**CHMP4C**	**Chromatin Modifying Protein 4C**	**0.303024394**
**CLCA2**	**Chloride Channel Accessory 2**	**0.308887166**
**CLIC4**	**Chloride Intracellular Channel 4**	**0.610508051**
**CLIP1**	**CAP-GLY Domain Containing Linker Protein 1**	**0.527130432**
**CLOCK**	**Clock Homolog (Mouse)**	**0.568629326**
**CLTC**	**Clathrin, Heavy Chain (Hc)**	**0.015644364**
**CMPK1**	**Cytidine Monophosphate (UMP-CMP) Kinase 1, Cytosolic**	**0.301625174**
**CNIH4**	**Cornichon Homolog 4 (Drosophila)**	**0.492505655**
**COPS2**	**COP9 Constitutive Photomorphogenic Homolog Subunit 2 (Arabidopsis)**	**0.144382973**
**CORO1C**	**Coronin, Actin Binding Protein, 1C**	**0.539970931**
**COX17**	**COX17 Cytochrome C Oxidase Assembly Homolog (S. Cerevisiae)**	**1.511555653**
**COX7B**	**Cytochrome C Oxidase Subunit Viib**	**0.524889903**
**CP110**	**CP110 Protein**	**0.520282214**
**CPOX**	**Coproporphyrinogen Oxidase**	**0.226361633**
**CRY1**	**Cryptochrome 1 (Photolyase-Like)**	**0.598409996**
**CTAGE6**	**CTAGE Family, Member 6**	**0.32859381**
**CUL4A**	**Cullin 4A**	**0.595244664**
**CXorf40B**	**Chromosome X Open Reading Frame 40A; Chromosome X Open Reading Frame 40B**	**0.508184627**
**CYP24A1**	**Cytochrome P450, Family 24, Subfamily A, Polypeptide 1**	**0.070662785**
**CYP51A1**	**Cytochrome P450, Family 51, Subfamily A, Polypeptide 1**	**0.070605006**
**CYTH2**	**Cytohesin 2**	**2.216600495**
**DAPP1**	**Dual Adaptor Of Phosphotyrosine And 3-Phosphoinositides**	**0.560721379**
**DBR1**	**Debranching Enzyme Homolog 1 (S. Cerevisiae)**	**0.534345485**
**DCTN4**	**Dynactin 4 (P62)**	**0.461618626**
**DCUN1D3**	**DCN1, Defective In Cullin Neddylation 1, Domain Containing 3 (S. Cerevisiae)**	**1.908573339**
**DDX17**	**DEAD (Asp-Glu-Ala-Asp) Box Polypeptide 17**	**0.351977262**
**DEK**	**DEK Oncogene**	**0.346863258**
**DLD**	**Dihydrolipoamide Dehydrogenase**	**0.465006354**
**DLG1**	**Discs, Large Homolog 1 (Drosophila)**	**0.355119032**
**DNAJC10**	**Dnaj (Hsp40) Homolog, Subfamily C, Member 10**	**0.447429614**
**DOPEY2**	**Dopey Family Member 2**	**0.436443393**
**DPYSL3**	**Dihydropyrimidinase-Like 3**	**2.028334346**
**DR1**	**Down-Regulator Of Transcription 1, TBP-Binding (Negative Cofactor 2)**	**0.518962794**

**DST**	**Dystonin**	**0.297412592**
**DTL**	**Denticleless Homolog (Drosophila)**	**0.284344431**
**DUSP6**	**Dual Specificity Phosphatase 6**	**0.542980598**
**EAF1**	**ELL Associated Factor 1**	**0.240749295**
**EAPP**	**E2F-Associated Phosphoprotein**	**0.415071517**
**ECHDC1**	**Enoyl Coenzyme A Hydratase Domain Containing 1**	**0.220817255**
**ECT2**	**Epithelial Cell Transforming Sequence 2 Oncogene**	**0.286367254**
**EDEM3**	**ER Degradation Enhancer, Mannosidase Alpha-Like 3**	**0.365874418**
**EED**	**Embryonic Ectoderm Development**	**0.138475525**
**EHD2**	**EH-Domain Containing 2**	**0.478068236**
**EHF**	**Ets Homologous Factor**	**1.999099264**
**EIF1AX**	**Eukaryotic Translation Initiation Factor 1A, X-Linked**	**0.21517204**
**EIF2B3**	**Eukaryotic Translation Initiation Factor 2B, Subunit 3 Gamma, 58kda**	**0.534188073**
**EIF2S1**	**Eukaryotic Translation Initiation Factor 2, Subunit 1 Alpha, 35kda**	**0.475790905**
**EIF2S2**	**Eukaryotic Translation Initiation Factor 2, Subunit 2 Beta, 38kda**	**1.811125677**
**EIF3A**	**Eukaryotic Translation Initiation Factor 3, Subunit A**	**0.297396859**
**EIF3C**	**Eukaryotic Translation Initiation Factor 3, Subunit C**	**0.225447112**
**ELOVL5**	**ELOVL Family Member 5, Elongation Of Long Chain Fatty Acids (FEN1/Elo2, SUR4/Elo3-Like, Yeast)**	**0.300879016**
**ERGIC2**	**ERGIC And Golgi 2**	**0.096228046**
**ETS1**	**V-Ets Erythroblastosis Virus E26 Oncogene Homolog 1 (Avian)**	**0.518697078**
**ETV4**	**Ets Variant 4**	**1.67270165**
**F11R**	**F11 Receptor**	**0.189040589**
**F3**	**Coagulation Factor III (Thromboplastin, Tissue Factor)**	**0.507397062**
**FAHD2A**	**Fumarylacetoacetate Hydrolase Domain Containing 2A**	**0.303951503**
**FAM103A1**	**Family With Sequence Similarity 103, Member A1**	**0.485511235**
**FAM104A**	**Family With Sequence Similarity 104, Member A**	**0.456888109**
**FAM10A4**	**Similar To Heat Shock 70kd Protein Binding Protein; Family With Sequence Similarity 10, Member A4 Pseudogene; Family With Sequence Similarity 10, Member A5 Pseudogene**	**0.347490127**
**FAM116A**	**Family With Sequence Similarity 116, Member A**	**0.263495701**
**FAM133B**	**Family With Sequence Similarity 133, Member B Pseudogene; Similar To FAM133B Protein; Family With Sequence Similarity 133, Member B**	**0.461175316**
**FAM135A**	**Family With Sequence Similarity 135, Member A**	**0.553824304**
**FAM162A**	**Family With Sequence Similarity 162, Member A**	**0.113883056**
**FAM179B**	**Family With Sequence Similarity 179, Member B**	**0.083768862**
**FAM49B**	**Family With Sequence Similarity 49, Member B**	**0.131799832**
**FAM84B**	**Family With Sequence Similarity 84, Member B**	**1.639021357**
**FAM8A1**	**Family With Sequence Similarity 8, Member A1**	**0.483323986**
**FAM98A**	**Family With Sequence Similarity 98, Member A**	**0.219492554**
**FANCI**	**Fanconi Anemia, Complementation Group I**	**0.362697384**

**FCHO2**	**FCH Domain Only 2**	**0.179138912**
**FDPS**	**Farnesyl Diphosphate Synthase (Farnesyl Pyrophosphate Synthetase, Dimethylallyltranstransferase, Geranyltranstransferase)**	**0.535883674**
**FEM1B**	**Fem-1 Homolog B (C. Elegans)**	**2.170755044**
**FH**	**Fumarate Hydratase**	**0.418548959**
**FKBP3**	**FK506 Binding Protein 3, 25kda**	**0.343357073**
**FLJ31306**	**Hypothetical LOC379025**	**0.577326432**
**FLJ35390**	**Hypothetical LOC255031**	**0.305768217**
**FLJ43681**	**Similar To 60S Ribosomal Protein L23a**	**0.146394587**
**FLJ44124**	**Hypothetical LOC641737**	**0.414685138**
**FNDC3A**	**Fibronectin Type III Domain Containing 3A**	**0.218908661**
**FOXQ1**	**Forkhead Box Q1**	**1.767075991**
**FRG1**	**FSHD Region Gene 1**	**0.355744115**
**FTHL11**	**Ferritin, Heavy Polypeptide-Like 11**	**0.210559425**
**FTHL12**	**Ferritin, Heavy Polypeptide-Like 12**	**0.160475986**
**FTHL2**	**Ferritin, Heavy Polypeptide-Like 2**	**0.169440946**
**FTHL3**	**Ferritin, Heavy Polypeptide 1; Ferritin, Heavy Polypeptide-Like 16; Similar To Ferritin, Heavy Polypeptide 1; Ferritin, Heavy Polypeptide-Like 3 Pseudogene**	**0.15793614**
**FTHL8**	**Ferritin, Heavy Polypeptide-Like 8**	**0.08989911**
**FXR2**	**Fragile X Mental Retardation, Autosomal Homolog 2**	**1.645484586**
**FYTTD1**	**Forty-Two-Three Domain Containing 1**	**0.376298344**
**FZD6**	**Frizzled Homolog 6 (Drosophila)**	**0.049175021**
**G3BP1**	**Gtpase Activating Protein (SH3 Domain) Binding Protein 1**	**0.476697791**
**GADD45GIP1**	**Growth Arrest And DNA-Damage-Inducible, Gamma Interacting Protein 1**	**0.181244957**
**GALNT1**	**UDP-N-Acetyl-Alpha-D-Galactosamine:Polypeptide N-Acetylgalactosaminyltransferase 13 (Galnac-T13); UDP-N-Acetyl-Alpha-D-Galactosamine:Polypeptide N-Acetylgalactosaminyltransferase 1 (Galnac-T1)**	**0.242730733**
**GALNT14**	**UDP-N-Acetyl-Alpha-D-Galactosamine:Polypeptide N-Acetylgalactosaminyltransferase 14 (Galnac-T14)**	**0.247320705**
**GCLC**	**Glutamate-Cysteine Ligase, Catalytic Subunit**	**0.432002774**
**GDI2**	**GDP Dissociation Inhibitor 2**	**0.579959904**
**GGH**	**Gamma-Glutamyl Hydrolase (Conjugase, Folylpolygammaglutamyl Hydrolase)**	**0.604981067**
**GJA1**	**Gap Junction Protein, Alpha 1, 43kda**	**0.29821623**
**GJB6**	**Gap Junction Protein, Beta 6, 30kda**	**0.433931358**
**GLRX2**	**Glutaredoxin 2**	**0.559284612**
**GMNN**	**Geminin, DNA Replication Inhibitor**	**0.246919507**
**GNB4**	**Guanine Nucleotide Binding Protein (G Protein), Beta Polypeptide 4**	**0.369353985**
**GOLGB1**	**Golgin B1, Golgi Integral Membrane Protein**	**0.506993314**
**GOLT1B**	**Golgi Transport 1 Homolog B (S. Cerevisiae)**	**0.364876395**
**GPAM**	**Glycerol-3-Phosphate Acyltransferase, Mitochondrial**	**0.516406308**
**GPR126**	**G Protein-Coupled Receptor 126**	**1.85390473**
**GTF2H2B**	**General Transcription Factor IIH, Polypeptide 2, 44kda;**	**0.38450185**

	**General Transcription Factor IIH, Polypeptide 2C; General Transcription Factor IIH, Polypeptide 2B; General Transcription Factor IIH, Polypeptide 2D**	
**HAT1**	**Histone Acetyltransferase 1**	**0.243148561**
**HBEGF**	**Heparin-Binding EGF-Like Growth Factor**	**1.507772468**
**HDGFRP3**	**Hepatoma-Derived Growth Factor, Related Protein 3**	**0.224243841**
**HDHD3**	**Haloacid Dehalogenase-Like Hydrolase Domain Containing 3**	**0.451969526**
**HEBP2**	**Heme Binding Protein 2**	**0.360955858**
**HIF1A**	**Hypoxia Inducible Factor 1, Alpha Subunit (Basic Helix-Loop-Helix Transcription Factor)**	**0.324408297**
**HMGB1L1**	**High-Mobility Group Box 1-Like 1**	**0.207008801**
**HMGB3**	**Similar To High Mobility Group Box 3; High-Mobility Group Box 3**	**1.910078078**
**HNRNPA3P1**	**Heterogeneous Nuclear Ribonucleoprotein A3 Pseudogene 1**	**0.343886384**
**HNRNPH3**	**Heterogeneous Nuclear Ribonucleoprotein H3 (2H9)**	**0.578435829**
**HNRPA1L-2**	**Heterogeneous Nuclear Ribonucleoprotein A1-Like 3; Similar To Heterogeneous Nuclear Ribonucleoprotein A1; Heterogeneous Nuclear Ribonucleoprotein A1 Pseudogene 2; Heterogeneous Nuclear Ribonucleoprotein A1; Heterogeneous Nuclear Ribonucleoprotein A1 Pseudogene**	**0.310333503**
**HS3ST1**	**Heparan Sulfate (Glucosamine) 3-O-Sulfotransferase 1**	**2.658779307**
**HSD17B1**	**Hydroxysteroid (17-Beta) Dehydrogenase 1**	**0.570047338**
**HSD17B2**	**Hydroxysteroid (17-Beta) Dehydrogenase 2**	**1.669447047**
**HSPA1A**	**Heat Shock 70kda Protein 1A; Heat Shock 70kda Protein 1B**	**0.09822051**
**HSPA4**	**Heat Shock 70kda Protein 4**	**0.519859531**
**IBTK**	**Inhibitor Of Bruton Agammaglobulinemia Tyrosine Kinase**	**0.296664618**
**IDI1**	**Isopentenyl-Diphosphate Delta Isomerase 1**	**0.273629482**
**IER3**	**Immediate Early Response 3**	**1.984686075**
**IER3IP1**	**Immediate Early Response 3 Interacting Protein 1**	**0.237435563**
**IFI27**	**Interferon, Alpha-Inducible Protein 27**	**1.558407935**
**IFNGR1**	**Interferon Gamma Receptor 1**	**1.550763011**
**IFT20**	**Intraflagellar Transport 20 Homolog (Chlamydomonas)**	**0.415491951**
**IFT74**	**Intraflagellar Transport 74 Homolog (Chlamydomonas)**	**0.3265505**
**IGFBP4**	**Insulin-Like Growth Factor Binding Protein 4**	**2.098727892**
**IL18**	**Interleukin 18 (Interferon-Gamma-Inducing Factor)**	**0.543008922**
**IL1A**	**Interleukin 1, Alpha**	**1.640185348**
**IL1B**	**Interleukin 1, Beta**	**1.844758129**
**IL1F9**	**Interleukin 1 Family, Member 9**	**7.782343631**
**IMMT**	**Inner Membrane Protein, Mitochondrial (Mitofilin)**	**0.596371026**
**INPP4B**	**Inositol Polyphosphate-4-Phosphatase, Type II, 105kda**	**0.592913737**
**INTS8**	**Integrator Complex Subunit 8**	**0.21752267**
**IQGAP1**	**IQ Motif Containing Gtpase Activating Protein 1**	**0.392781111**
**ISOC1**	**Isochorismatase Domain Containing 1**	**0.571547199**
**ITGA6**	**Integrin, Alpha 6**	**0.193571039**
**JUN**	**Jun Oncogene**	**1.738365679**
**JUND**	**Jun D Proto-Oncogene**	**1.720596384**

**KBTBD7**	**Kelch Repeat And BTB (POZ) Domain Containing 7**	**0.065623411**
**KCNK1**	**Potassium Channel, Subfamily K, Member 1**	**0.59726315**
**KCNS3**	**Potassium Voltage-Gated Channel, Delayed-Rectifier, Subfamily S, Member 3**	**0.602139396**
**KHNYN**	**Kiaa0323**	**1.790685669**
**KIAA0100**	**Kiaa0100**	**0.171189492**
**KIF16B**	**Kinesin Family Member 16B**	**0.412793817**
**KITLG**	**KIT Ligand**	**0.532878119**
**KLF5**	**Kruppel-Like Factor 5 (Intestinal)**	**0.007540615**
**KLHL2**	**Kelch-Like 2, Mayven (Drosophila)**	**0.286192039**
**KPNA2**	**Karyopherin Alpha 2 (RAG Cohort 1, Importin Alpha 1); Karyopherin Alpha-2 Subunit Like**	**0.412574533**
**KRCC1**	**Lysine-Rich Coiled-Coil 1**	**0.426870376**
**KRT7**	**Keratin 7**	**2.755440998**
**KRT8**	**Keratin 8 Pseudogene 9; Similar To Keratin 8; Keratin 8**	**1.820185493**
**KTN1**	**Kinectin 1 (Kinesin Receptor)**	**0.578449499**
**KYNU**	**Kynureninase (L-Kynurenine Hydrolase)**	**1.920123103**
**LAPTM4A**	**Lysosomal Protein Transmembrane 4 Alpha**	**0.542857689**
**LARP4**	**La Ribonucleoprotein Domain Family, Member 4**	**0.250320211**
**LCLAT1**	**Lysocardiolipin Acyltransferase 1**	**0.580250266**
**LCOR**	**Ligand Dependent Nuclear Receptor Corepressor**	**0.48387851**
**LDHB**	**Lactate Dehydrogenase B**	**0.502886246**
**LGALS3**	**Lectin, Galactoside-Binding, Soluble, 3**	**0.088575631**
**LGALS8**	**Lectin, Galactoside-Binding, Soluble, 8**	**0.56291483**
**LIMS1**	**LIM And Senescent Cell Antigen-Like Domains 1**	**0.376184048**
**LIN7C**	**Lin-7 Homolog C (C. Elegans)**	**0.026250711**
**LRRC57**	**Leucine Rich Repeat Containing 57**	**0.516104664**
**LRRC59**	**Leucine Rich Repeat Containing 59**	**0.337915919**
**LSM6**	**LSM6 Homolog, U6 Small Nuclear RNA Associated (S. Cerevisiae)**	**0.151203718**
**MAK16**	**MAK16 Homolog (S. Cerevisiae)**	**0.34510299**
**MAN2A1**	**Mannosidase, Alpha, Class 2A, Member 1**	**0.481241497**
**MAP3K1**	**Mitogen-Activated Protein Kinase Kinase Kinase 1**	**0.605499629**
**MBD1**	**Methyl-Cpg Binding Domain Protein 1**	**1.664483473**
**MBNL2**	**Muscleblind-Like 2 (Drosophila)**	**0.362948989**
**MCTS1**	**Malignant T Cell Amplified Sequence 1**	**0.094512425**
**ME1**	**Malic Enzyme 1, NADP(+)-Dependent, Cytosolic**	**0.325831225**
**MET**	**Met Proto-Oncogene (Hepatocyte Growth Factor Receptor)**	**0.418234024**
**MEX3C**	**Mex-3 Homolog C (C. Elegans)**	**0.286953554**
**MFSD11**	**Major Facilitator Superfamily Domain Containing 11**	**0.421823169**
**MINPP1**	**Multiple Inositol Polyphosphate Histidine Phosphatase, 1**	**0.241162789**
**MKL2**	**MKL/Myocardin-Like 2**	**0.106839901**
**MMP1**	**Matrix Metallopeptidase 1 (Interstitial Collagenase)**	**0.13679624**
**MNAT1**	**Menage A Trois Homolog 1, Cyclin H Assembly Factor (Xenopus Laevis)**	**0.340016358**
**MOBKL1A**	**MOB1, Mps One Binder Kinase Activator-Like 1A (Yeast)**	**0.271112658**

**MOBKL1B**	**MOB1, Mps One Binder Kinase Activator-Like 1B (Yeast)**	**0.479291167**
**MORF4L1**	**Mortality Factor 4; Mortality Factor 4 Like 1**	**0.472746945**
**MPHOSPH6**	**M-Phase Phosphoprotein 6**	**0.323160328**
**MRP63**	**Mitochondrial Ribosomal Protein 63**	**0.57949402**
**MRPL1**	**Mitochondrial Ribosomal Protein L1**	**0.411868352**
**MRPL10**	**Mitochondrial Ribosomal Protein L10**	**0.409928232**
**MRPL15**	**Mitochondrial Ribosomal Protein L15**	**0.287339226**
**MRPL19**	**Mitochondrial Ribosomal Protein L19**	**0.199634623**
**MRPL48**	**Mitochondrial Ribosomal Protein L48**	**0.480290158**
**MSH6**	**Muts Homolog 6 (E. Coli)**	**0.602195021**
**MSTO1**	**Misato Homolog 1 (Drosophila)**	**0.223327103**
**MT1G**	**Metallothionein 1G**	**0.375304599**
**MTDH**	**Metadherin**	**0.393303705**
**MTF2**	**Metal Response Element Binding Transcription Factor 2**	**0.139955739**
**MTMR6**	**Myotubularin Related Protein 6**	**0.299644341**
**MTPN**	**Myotrophin; Leucine Zipper Protein 6**	**0.523093132**
**MYO10**	**Myosin X**	**0.175908597**
**MYO1B**	**Myosin IB**	**0.483186409**
**MYO5A**	**Myosin VA (Heavy Chain 12, Myoxin)**	**0.524355341**
**NACAP1**	**Nascent-Polypeptide-Associated Complex Alpha Polypeptide Pseudogene 1**	**0.05834918**
**NARS**	**Asparaginyl-Trna Synthetase**	**0.429246088**
**NAV3**	**Neuron Navigator 3; Similar To Neuron Navigator 3**	**1.629897814**
**NBPF8**	**Neuroblastoma Breakpoint Family, Member 15; Neuroblastoma Breakpoint Family, Member 14; Neuroblastoma Breakpoint Family, Member 9; Neuroblastoma Breakpoint Family, Member 11; Neuroblastoma Breakpoint Family, Member 11-Like; Neuroblastoma Breakpoint Family, Member 10; Neuroblastoma Breakpoint Family, Member 12; Neuroblastoma Breakpoint Family, Member 20; Neuroblastoma Breakpoint Family, Member 1; KIAA1245; Neuroblastoma Breakpoint Family, Member 8**	**0.324201916**
**NDC80**	**NDC80 Homolog, Kinetochore Complex Component (S. Cerevisiae)**	**0.239886967**
**NDUFAB1**	**NADH Dehydrogenase (Ubiquinone) 1, Alpha/Beta Subcomplex, 1, 8kda**	**0.475542826**
**NDUFB10**	**NADH Dehydrogenase (Ubiquinone) 1 Beta Subcomplex, 10, 22kda**	**0.550325358**
**NDUFB9**	**NADH Dehydrogenase (Ubiquinone) 1 Beta Subcomplex, 9, 22kda**	**0.23944357**
**NEK1**	**NIMA (Never In Mitosis Gene A)-Related Kinase 1**	**0.540052268**
**NEK2**	**NIMA (Never In Mitosis Gene A)-Related Kinase 2**	**0.412903278**
**NMD3**	**NMD3 Homolog (S. Cerevisiae)**	**0.453167622**
**NMT2**	**N-Myristoyltransferase 2**	**0.432364083**
**NNT**	**Nicotinamide Nucleotide Transhydrogenase**	**0.510438471**
**NOP56**	**NOP56 Ribonucleoprotein Homolog (Yeast)**	**0.550114978**
**NR2F2**	**Nuclear Receptor Subfamily 2, Group F, Member 2**	**0.479682246**

**NT5E**	**5'-Nucleotidase, Ecto (CD73)**	**0.492775754**
**NUCB2**	**Nucleobindin 2**	**0.313516326**
**NUDCD1**	**Nudc Domain Containing 1**	**0.089256272**
**NUDCD2**	**Nudc Domain Containing 2**	**0.328290847**
**NUDT15**	**Nudix (Nucleoside Diphosphate Linked Moiety X)-Type Motif 15**	**0.604549631**
**NUP54**	**Nucleoporin 54kda**	**0.440154926**
**OSBPL11**	**Oxysterol Binding Protein-Like 11**	**0.235608426**
**OSBPL8**	**Oxysterol Binding Protein-Like 8**	**0.314812318**
**OTUD6B**	**OTU Domain Containing 6B**	**0.574584971**
**P4HA1**	**Prolyl 4-Hydroxylase, Alpha Polypeptide I**	**0.343255951**
**P704P**	**Prostate-Specific P704P**	**0.24755591**
**PCDHB19P**	**Protocadherin Beta 19 Pseudogene**	**0.395295412**
**PCNA**	**Proliferating Cell Nuclear Antigen**	**0.316369001**
**PDGFC**	**Platelet Derived Growth Factor C**	**0.339491871**
**PDIA3P**	**Protein Disulfide Isomerase Family A, Member 3 Pseudogene**	**0.271304568**
**PERP**	**PERP, TP53 Apoptosis Effector**	**0.137049865**
**PGD**	**Phosphogluconate Dehydrogenase**	**1.672105674**
**PGRMC2**	**Progesterone Receptor Membrane Component 2**	**0.520988539**
**PHAX**	**Phosphorylated Adaptor For RNA Export**	**0.592099809**
**PHF23**	**PHD Finger Protein 23**	**0.512266311**
**PHLDB2**	**Pleckstrin Homology-Like Domain, Family B, Member 2; Phosphatidylinositol-Specific Phospholipase C, X Domain Containing 2**	**0.293278891**
**PIGH**	**Phosphatidylinositol Glycan Anchor Biosynthesis, Class H**	**0.399620363**
**PIH1D1**	**PIH1 Domain Containing 1**	**1.590694364**
**PIK3C3**	**Phosphoinositide-3-Kinase, Class 3**	**0.2749049**
**PKN2**	**Protein Kinase N2**	**0.464797203**
**PLA2G2D**	**Phospholipase A2, Group IID**	**0.26860001**
**PLAA**	**Phospholipase A2-Activating Protein**	**0.431262352**
**PLAUR**	**Plasminogen Activator, Urokinase Receptor**	**1.867191412**
**PLEKHA5**	**Pleckstrin Homology Domain Containing, Family A Member 5**	**0.081179441**
**PLIN5**	**Lipid Storage Droplet Protein 5**	**0.365116868**
**PMAIP1**	**Phorbol-12-Myristate-13-Acetate-Induced Protein 1**	**0.197976665**
**PNPLA8**	**Patatin-Like Phospholipase Domain Containing 8**	**0.225219595**
**POLR2J2**	**Polymerase (RNA) II (DNA Directed) Polypeptide J3; Polymerase (RNA) II (DNA Directed) Polypeptide J2**	**0.404883145**
**POLR2J3**	**Polymerase (RNA) II (DNA Directed) Polypeptide J3; Polymerase (RNA) II (DNA Directed) Polypeptide J2**	**0.45666767**
**POLR2K**	**Polymerase (RNA) II (DNA Directed) Polypeptide K, 7.0kda**	**0.018046235**
**POLR3F**	**Polymerase (RNA) III (DNA Directed) Polypeptide F, 39 Kda**	**0.520118961**
**POP4**	**Processing Of Precursor 4, Ribonuclease P/MRP Subunit (S. Cerevisiae)**	**0.438679117**
**PPA2**	**Pyrophosphatase (Inorganic) 2**	**0.429303571**

**PPIA**	**Similar To Trimcyp; Peptidylprolyl Isomerase A (Cyclophilin A); Peptidylprolyl Isomerase A (Cyclophilin A)-Like 3**	**0.433412091**
**PPIB**	**Peptidylprolyl Isomerase B (Cyclophilin B)**	**0.029195178**
**PPM1K**	**Protein Phosphatase 1K (PP2C Domain Containing)**	**0.402163345**
**PPP1R12A**	**Protein Phosphatase 1, Regulatory (Inhibitor) Subunit 12A**	**0.228550447**
**PPT1**	**Palmitoyl-Protein Thioesterase 1**	**0.465832021**
**PRKCI**	**Protein Kinase C, Iota**	**0.418015375**
**PRKRA**	**Protein Kinase, Interferon-Inducible Double Stranded RNA Dependent Activator**	**0.308411558**
**PRPF18**	**PRP18 Pre-Mrna Processing Factor 18 Homolog (S. Cerevisiae)**	**0.306297403**
**PRRC1**	**Proline-Rich Coiled-Coil 1**	**0.406718234**
**PRRG4**	**Proline Rich Gla (G-Carboxyglutamic Acid) 4 (Transmembrane)**	**0.463769997**
**PSMC5**	**Proteasome (Prosome, Macropain) 26S Subunit, Atpase, 5**	**0.606965656**
**PTGES3**	**Prostaglandin E Synthase 3 (Cytosolic)**	**0.118497593**
**PTMA**	**Hypothetical LOC728026; Prothymosin, Alpha; Hypothetical Gene Supported By BC013859; Prothymosin, Alpha Pseudogene 4 (Gene Sequence 112)**	**0.075025657**
**PTPLAD2**	**Protein Tyrosine Phosphatase-Like A Domain Containing 2**	**0.238337742**
**PTPLB**	**Protein Tyrosine Phosphatase-Like (Proline Instead Of Catalytic Arginine), Member B**	**0.433034452**
**PXN**	**Paxillin**	**0.589190639**
**PYGL**	**Phosphorylase, Glycogen, Liver**	**0.585773856**
**PYROXD1**	**Pyridine Nucleotide-Disulphide Oxidoreductase Domain 1**	**0.008004783**
**QPRT**	**Quinolinate Phosphoribosyltransferase**	**1.770923878**
**RAB13**	**RAB13, Member RAS Oncogene Family; Similar To Hcg24991**	**0.547514167**
**RAB18**	**RAB18, Member RAS Oncogene Family**	**0.410036186**
**RAB38**	**RAB38, Member RAS Oncogene Family**	**1.820841155**
**RAB8B**	**RAB8B, Member RAS Oncogene Family**	**0.405148911**
**RABL2A**	**RAB, Member Of RAS Oncogene Family-Like 2A**	**0.519508048**
**RAP1B**	**RAP1B, Member Of RAS Oncogene Family**	**0.22672955**
**RAP1BL**	**Hcg1757335**	**0.118245106**
**RAP2C**	**RAP2C, Member Of RAS Oncogene Family**	**0.523444595**
**RBBP7**	**Retinoblastoma Binding Protein 7**	**0.50321874**
**RBM34**	**RNA Binding Motif Protein 34**	**0.348422285**
**RBM42**	**RNA Binding Motif Protein 42**	**1.533654643**
**RBM47**	**RNA Binding Motif Protein 47**	**1.824456649**
**RBM7**	**RNA Binding Motif Protein 7**	**0.223957489**
**RELL1**	**RELT-Like 1**	**0.20115905**
**RHBDL2**	**Rhomboid, Veinlet-Like 2 (Drosophila)**	**0.267895551**
**RIF1**	**RAP1 Interacting Factor Homolog (Yeast)**	**0.336943177**
**RILPL2**	**Rab Interacting Lysosomal Protein-Like 2**	**1.662762125**
**RNASEH2B**	**Ribonuclease H2, Subunit B**	**0.134005812**
**RNPC3**	**RNA-Binding Region (RNP1, RRM) Containing 3**	**0.560207909**

**RPF1**	**Brix Domain Containing 5**	**0.515632312**
**RPL12P6**	**Ribosomal Protein L12 Pseudogene 2; Ribosomal Protein L12 Pseudogene 32; Ribosomal Protein L12 Pseudogene 35; Ribosomal Protein L12 Pseudogene 19; Ribosomal Protein L12 Pseudogene 6; Ribosomal Protein L12; Ribosomal Protein L12 Pseudogene 14**	**0.551081488**
**RPL14**	**Ribosomal Protein L14**	**0.154624118**
**RPL23**	**Ribosomal Protein L23 Pseudogene 6; Ribosomal Protein L23**	**0.125555268**
**RPL23AP53**	**Ribosomal Protein L23a Pseudogene 53**	**0.466993426**
**RPL5**	**Ribosomal Protein L5 Pseudogene 34; Ribosomal Protein L5 Pseudogene 1; Ribosomal Protein L5**	**0.359161407**
**RPL7**	**Ribosomal Protein L7 Pseudogene 26; Ribosomal Protein L7 Pseudogene 16; Ribosomal Protein L7; Ribosomal Protein L7 Pseudogene 32; Ribosomal Protein L7 Pseudogene 23; Ribosomal Protein L7 Pseudogene 24; Ribosomal Protein L7 Pseudogene 20**	**0.243044989**
**RPN2**	**Ribophorin II**	**0.25379088**
**RPS28**	**Ribosomal Protein S28 Pseudogene 6; Ribosomal Protein S28 Pseudogene 9; Ribosomal Protein S28**	**0.145942225**
**RPS3A**	**Ribosomal Protein S3A Pseudogene 5; Ribosomal Protein S3a Pseudogene 47; Ribosomal Protein S3a Pseudogene 49; Ribosomal Protein S3A; Hypothetical LOC100131699; Hypothetical LOC100130107**	**0.575903294**
**RPS4Y2**	**Ribosomal Protein S4, Y-Linked 2**	**0.17791788**
**RPS6KC1**	**Ribosomal Protein S6 Kinase, 52kda, Polypeptide 1**	**0.591524514**
**RPS6P1**	**Ribosomal Protein S6 Pseudogene 25; Ribosomal Protein S6; Ribosomal Protein S6 Pseudogene 1**	**0.383929183**
**RRN3**	**RRN3 RNA Polymerase I Transcription Factor Homolog (S. Cerevisiae)**	**0.456209854**
**SACM1L**	**SAC1 Suppressor Of Actin Mutations 1-Like (Yeast)**	**0.182520283**
**SACS**	**Spastic Ataxia Of Charlevoix-Saguenay (Sacsin)**	**0.091032256**
**SAP30**	**Sin3A-Associated Protein, 30kda**	**0.27190006**
**SAP30L**	**SAP30-Like**	**0.577234866**
**SAR1A**	**SAR1 Homolog A (S. Cerevisiae)**	**0.319623204**
**SAV1**	**Salvador Homolog 1 (Drosophila)**	**0.256852251**
**SBDS**	**Shwachman-Bodian-Diamond Syndrome Pseudogene; Shwachman-Bodian-Diamond Syndrome**	**0.126770293**
**SC4MOL**	**Sterol-C4-Methyl Oxidase-Like**	**0.466771995**
**SEC24A**	**SEC24 Family, Member A (S. Cerevisiae)**	**0.147938166**
**SEC24D**	**SEC24 Family, Member D (S. Cerevisiae)**	**0.19734612**
**SEMA3C**	**Sema Domain, Immunoglobulin Domain (Ig), Short Basic Domain, Secreted, (Semaphorin) 3C**	**0.067574799**
**SERINC1**	**Serine Incorporator 1**	**0.599550895**
**SETD6**	**SET Domain Containing 6**	**0.440984679**
**SFPQ**	**Splicing Factor Proline/Glutamine-Rich (Polypyrimidine Tract Binding Protein Associated)**	**0.378773173**
**SFRS11**	**Splicing Factor, Arginine/Serine-Rich 11**	**0.066319712**
**SFRS12**	**Splicing Factor, Arginine/Serine-Rich 12**	**0.372408253**
**SGK1**	**Serum/Glucocorticoid Regulated Kinase 1**	**1.864963276**

**SH3YL1**	**SH3 Domain Containing, Ysc84-Like 1 (S. Cerevisiae)**	**0.174273134**
**SHOC2**	**Soc-2 Suppressor Of Clear Homolog (C. Elegans)**	**0.566097766**
**SKA2**	**Family With Sequence Similarity 33, Member A; Similar To Spindle And Kinetochore-Associated Protein 2**	**0.514542278**
**SKIV2L2**	**Superkiller Viralicidic Activity 2-Like 2 (S. Cerevisiae)**	**0.51157187**
**SLBP**	**Stem-Loop Binding Protein**	**0.557806374**
**SLC11A2**	**Solute Carrier Family 11 (Proton-Coupled Divalent Metal Ion Transporters), Member 2**	**1.700400054**
**SLC38A2**	**Solute Carrier Family 38, Member 2**	**0.463555413**
**SLC38A6**	**Solute Carrier Family 38, Member 6**	**0.257206774**
**SLK**	**STE20-Like Kinase (Yeast)**	**0.46249861**
**SMARCA1**	**SWI/SNF Related, Matrix Associated, Actin Dependent Regulator Of Chromatin, Subfamily A, Member 1**	**0.543585807**
**SMARCA2**	**SWI/SNF Related, Matrix Associated, Actin Dependent Regulator Of Chromatin, Subfamily A, Member 2**	**0.448461114**
**SMARCA5**	**SWI/SNF Related, Matrix Associated, Actin Dependent Regulator Of Chromatin, Subfamily A, Member 5**	**0.320480122**
**SMCR5**	**Smith-Magenis Syndrome Chromosome Region, Candidate 5 (Non-Protein Coding)**	**0.335780961**
**SMNDC1**	**Survival Motor Neuron Domain Containing 1**	**0.404014875**
**SNAPC5**	**Small Nuclear RNA Activating Complex, Polypeptide 5, 19kda**	**0.074204052**
**SNHG8**	**Small Nucleolar RNA Host Gene 8 (Non-Protein Coding)**	**0.130911189**
**SNORD36A**	**Small Nucleolar RNA, C/D Box 36C; Small Nucleolar RNA, C/D Box 36B; Small Nucleolar RNA, C/D Box 36A**	**0.275090084**
**SNORD80**	**Small Nucleolar RNA, C/D Box 80**	**0.12696934**
**SNRNP35**	**ATP-Binding Cassette, Sub-Family B (MDR/TAP), Member 5; Small Nuclear Ribonucleoprotein 35kda (U11/U12)**	**0.566863355**
**SNX10**	**Sorting Nexin 10**	**0.154243778**
**SNX14**	**Sorting Nexin 14**	**0.501471023**
**SNX24**	**Sorting Nexin 24**	**0.392365848**
**SNX4**	**Sorting Nexin 4**	**0.562509388**
**SNX7**	**Sorting Nexin 7**	**0.423167784**
**SORD**	**Sorbitol Dehydrogenase**	**0.602793371**
**SOX7**	**SRY (Sex Determining Region Y)-Box 7**	**1.803344966**
**SP3**	**Sp3 Transcription Factor**	**0.488114581**
**SPIN4**	**Spindlin Family, Member 4**	**0.396543218**
**SPOPL**	**Speckle-Type POZ Protein-Like**	**0.151397448**
**SPRR2A**	**Small Proline-Rich Protein 2A**	**4.54542872**
**SRP72**	**Signal Recognition Particle 72kda**	**0.577425233**
**SS18**	**Synovial Sarcoma Translocation, Chromosome 18**	**0.556873412**
**SSTR2**	**Somatostatin Receptor 2**	**0.313359848**
**SUB1**	**SUB1 Homolog (S. Cerevisiae)**	**0.549705585**
**SUMO1P3**	**SMT3 Suppressor Of Mif Two 3 Homolog 1 (S. Cerevisiae); SUMO1 Pseudogene 3**	**0.390575847**
**SYAP1**	**Synapse Associated Protein 1, SAP47 Homolog (Drosophila)**	**0.439021367**
**TAF1D**	**TATA Box Binding Protein (TBP)-Associated Factor, RNA Polymerase I, D, 41kda; Small Nucleolar RNA, H/ACA Box**	**0.192075709**

	**32; Small Nucleolar RNA, H/ACA Box 25**	
**TAF9**	**TAF9 RNA Polymerase II, TATA Box Binding Protein (TBP)-Associated Factor, 32kda**	**0.16313925**
**TATDN1**	**Tatd Dnase Domain Containing 1**	**0.520179025**
**TBC1D14**	**TBC1 Domain Family, Member 14**	**1.628308247**
**TBC1D9**	**TBC1 Domain Family, Member 9 (With GRAM Domain)**	**0.425681783**
**TC2N**	**Tandem C2 Domains, Nuclear**	**0.103077526**
**TCP1**	**Hypothetical Gene Supported By BC000665; T-Complex 1**	**0.316257372**
**TDRD1**	**Tudor Domain Containing 1**	**0.330867075**
**TEAD3**	**TEA Domain Family Member 3**	**0.432454195**
**TFPI2**	**Tissue Factor Pathway Inhibitor 2**	**0.174062888**
**TLK1**	**Tousled-Like Kinase 1**	**0.493824896**
**TM2D1**	**TM2 Domain Containing 1**	**0.551567207**
**TM9SF2**	**Transmembrane 9 Superfamily Member 2**	**0.453827492**
**TMED10P**	**Transmembrane Emp24-Like Trafficking Protein 10 (Yeast) Pseudogene**	**0.596286479**
**TMED7**	**Transmembrane Emp24 Protein Transport Domain Containing 7; Toll-Like Receptor Adaptor Molecule 2**	**0.56382006**
**TMEM106B**	**Transmembrane Protein 106B**	**0.594502865**
**TMEM128**	**Transmembrane Protein 128**	**0.40399747**
**TMEM14B**	**Transmembrane Protein 14D; Transmembrane Protein 14B**	**0.560722112**
**TMEM154**	**Transmembrane Protein 154**	**0.450141426**
**TMEM184B**	**Transmembrane Protein 184B**	**1.609248758**
**TMEM184C**	**Transmembrane Protein 184C**	**0.475232857**
**TMEM199**	**Transmembrane Protein 199**	**0.575451893**
**TMEM200A**	**Transmembrane Protein 200A**	**0.343004914**
**TMEM30A**	**Transmembrane Protein 30A**	**0.53659605**
**TMEM45A**	**Transmembrane Protein 45A**	**0.387157876**
**TMEM55B**	**Transmembrane Protein 55B**	**1.807890439**
**TMSL3**	**Thymosin-Like 3**	**0.180517295**
**TOB1**	**Transducer Of ERBB2, 1**	**1.614041222**
**TOP1P2**	**Topoisomerase (DNA) I Pseudogene 2**	**0.070186844**
**TOPBP1**	**Topoisomerase (DNA) II Binding Protein 1**	**0.592269153**
**TPBG**	**Trophoblast Glycoprotein**	**0.294456871**
**TRAPPC4**	**Trafficking Protein Particle Complex 4**	**1.578310366**
**TRIM56**	**Tripartite Motif-Containing 56**	**0.469924241**
**TRIP12**	**Thyroid Hormone Receptor Interactor 12**	**0.28438912**
**TRMT6**	**Trna Methyltransferase 6 Homolog (S. Cerevisiae)**	**0.166588215**
**TUBA3D**	**Tubulin, Alpha 3d; Tubulin, Alpha 3c**	**0.203837563**
**TUBA4A**	**Tubulin, Alpha 4a**	**0.217925351**
**TUBB4Q**	**Tubulin, Beta Polypeptide 4, Member Q**	**0.275689888**
**TWF1**	**Twinfilin, Actin-Binding Protein, Homolog 1 (Drosophila)**	**0.347013434**
**TWSG1**	**Twisted Gastrulation Homolog 1 (Drosophila)**	**0.552918771**
**TXNDC9**	**Thioredoxin Domain Containing 9**	**0.196960887**
**TXNL1**	**Thioredoxin-Like 1**	**0.042632859**
**UAP1**	**UDP-N-Acteylglucosamine Pyrophosphorylase 1**	**0.268173063**

**UBA6**	**Ubiquitin-Like Modifier Activating Enzyme 6**	**0.227648908**
**UBE4A**	**Ubiquitination Factor E4A (UFD2 Homolog, Yeast)**	**0.265562967**
**UBLCP1**	**Ubiquitin-Like Domain Containing CTD Phosphatase 1**	**0.302304945**
**UBQLN2**	**Ubiquilin 2**	**0.491142755**
**UEVLD**	**UEV And Lactate/Malate Dehyrogenase Domains**	**0.524074128**
**UGDH**	**UDP-Glucose Dehydrogenase**	**0.531819335**
**UQCRC2**	**Ubiquinol-Cytochrome C Reductase Core Protein II**	**0.41133803**
**UQCRHL**	**Ubiquinol-Cytochrome C Reductase Hinge Protein-Like; Ubiquinol-Cytochrome C Reductase Hinge Protein**	**0.333132743**
**USP33**	**Ubiquitin Specific Peptidase 33**	**0.586350168**
**USP49**	**Ubiquitin Specific Peptidase 49**	**0.192614394**
**USP8**	**Ubiquitin Specific Peptidase 8**	**0.496552616**
**UTP3**	**UTP3, Small Subunit (SSU) Processome Component, Homolog (S. Cerevisiae)**	**0.118254446**
**VPS37A**	**Vacuolar Protein Sorting 37 Homolog A (S. Cerevisiae)**	**0.367440149**
**VRK2**	**Vaccinia Related Kinase 2**	**0.290947804**
**VTI1B**	**Vesicle Transport Through Interaction With T-Snares Homolog 1B (Yeast)**	**0.417896003**
**WRB**	**Tryptophan Rich Basic Protein**	**0.358618949**
**WSB2**	**WD Repeat And SOCS Box-Containing 2**	**0.10181075**
**XG**	**Xg Blood Group**	**0.182053573**
**YAP1**	**Yes-Associated Protein 1, 65kda**	**0.330270054**
**YEATS4**	**YEATS Domain Containing 4**	**0.504532461**
**YES1**	**V-Yes-1 Yamaguchi Sarcoma Viral Oncogene Homolog 1**	**0.216618348**
**YIPF4**	**Yip1 Domain Family, Member 4**	**0.441993632**
**YIPF5**	**Yip1 Domain Family, Member 5**	**0.226068654**
**YWHAG**	**Tyrosine 3-Monooxygenase/Tryptophan 5-Monooxygenase Activation Protein, Gamma Polypeptide**	**0.321469626**
**ZBTB8A**	**Zinc Finger And BTB Domain Containing 8A**	**0.118228539**
**ZC3H11B**	**Zinc Finger CCCH-Type Containing 11B Pseudogene**	**0.367905589**
**ZC3H12C**	**Zinc Finger CCCH-Type Containing 12C**	**2.262441704**
**ZFP36L1**	**Zinc Finger Protein 36, C3H Type-Like 1**	**0.123172907**
**ZFP36L2**	**Zinc Finger Protein 36, C3H Type-Like 2**	**1.572473511**
**ZNF12**	**Postmeiotic Segregation Increased 2-Like 3; Zinc Finger Protein 12**	**0.199032591**
**ZNF146**	**Zinc Finger Protein 146**	**0.380049685**
**ZNF281**	**Zinc Finger Protein 281**	**0.334948708**
**ZNF398**	**Zinc Finger Protein 398**	**0.313576047**
**ZNF486**	**Zinc Finger Protein 486**	**0.336385117**
**ZNF680**	**Zinc Finger Protein 680**	**0.334297369**
**ZNF738**	**Zinc Finger Protein 738**	**0.271961985**
**ZNF786**	**Zinc Finger Protein 786**	**0.492819711**

Table S3 - Gene Array List – The list of 528 differentially expressed genes (58 upregulated and 470 downregulated) post IL- 17A stimulation in primary keratinocytes.

Supplementary Figure S1

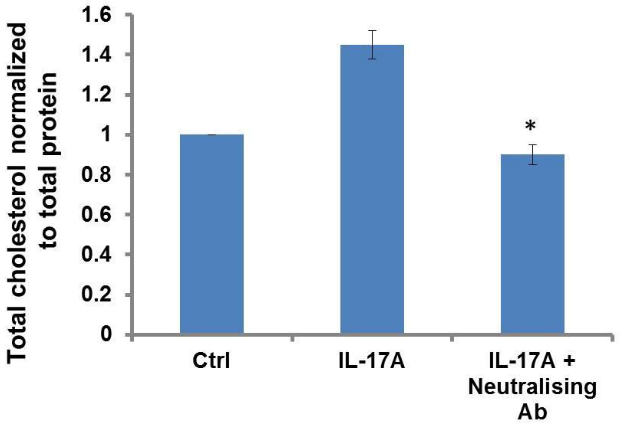

Supplementary Fig. S1 – IL-17A signaling was inhibited by pre-incubating the cytokine with IL-17A neutralising antibody (0.1ug/ml) for 30 mins. Total cholesterol content was measured in HaCaT cells stimulated with IL-17A or (IL-17A + Neutralising antibody complex) for 6h. * indicates p-value (≤ 0.05) in comparison to IL-17A stimulated cells.

Supplementary Figure S2 –

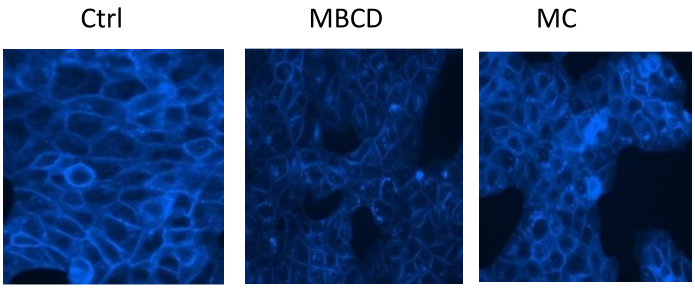

Supplementary Fig. S2 - HaCaT cells were treated with MBCD for cholesterol depletion and MBCD- cholesterol complex for cholesterol replenishment. Cells were stained by filipin for 1h at room temperature and visualized under fluorescence microscope.

Supplementary Figure S3

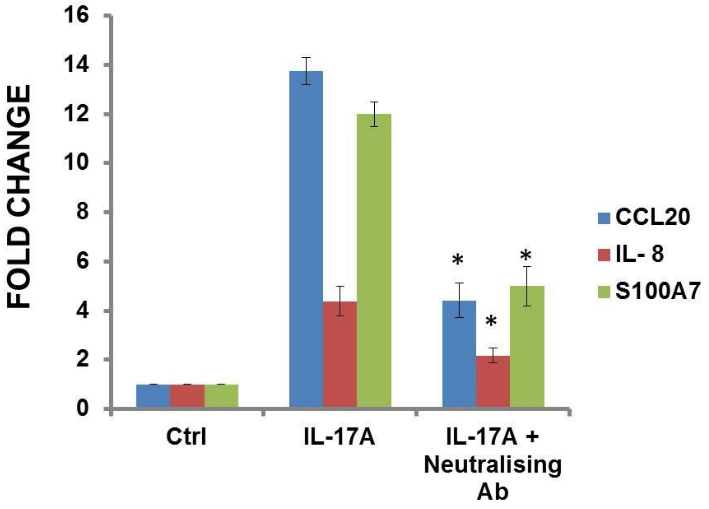

Supplementary Fig. S3 – Inhibiting IL-17A signaling by neutralizing antibody (0.1ug/ml) reduced the expression of CCL20, IL-8 and S100A7 compared to IL-17A stimulated HaCaT cells as measured by Q-RT PCR. * indicates p-value (≤ 0.05) in comparison to IL-17A stimulated cells.
